# From guideline to practice: three years of ICH S11 insights and recommendations

**DOI:** 10.3389/fmed.2025.1537001

**Published:** 2025-02-10

**Authors:** Diana Tavares, Hsiao-Tzu Chien, Maria Elzbieta Sheean, Peter Theunissen, Peter van Meer, Karen Van Malderen

**Affiliations:** ^1^Paediatric Medicines Office, European Medicines Agency, Amsterdam, Netherlands; ^2^Medicines Evaluation Board, Utrecht, Netherlands; ^3^Radboud University Medical Center, Nijmegen, Netherlands; ^4^Department of Pharmaceutics, Utrecht Institute of Pharmaceutical Sciences, Utrecht University, Utrecht, Netherlands; ^5^Federal Agency for Medicines and Health Products, Brussels, Belgium

**Keywords:** ICH S11, weight-of-evidence approach, juvenile animal study, Paediatric Investigation Plan, 3Rs, EMA

## Abstract

Juvenile Animal Studies (JAS) may be warranted to ensure the safe clinical use of medicines for children. The ICH S11 guideline was developed to guide the need for and design of JAS, and proposes a weight-of-evidence (WoE) approach. We evaluated how the introduction of the guideline shaped the non-clinical strategy for paediatric medicines in the European Union. Our review included Paediatric Investigation Plans (PIPs) for 127 products approved between 2020 and 2023, along with the associated regulatory assessment and final non-clinical plans. Although in 12 of selected PIPs a JAS was already ongoing or completed at the time of submission, in all other cases (115/127), the PIP was submitted before the initiation of JAS. In 75% (86/115) of these procedures the discussions based on the ICH S11 WoE approach led to an agreement on the proposed non-clinical strategy. In approximately a quarter of PIPs, there was disagreement on the outcome of the WoE analysis leading to the addition (3%), modification (10%), or removal (11%) of JAS. Our review indicates that the implementation of ICH S11 facilitates science-driven discussions about the necessity and design of JAS within the broader non-clinical strategy. A thorough consideration of developmental aspects of the product’s pharmacological target, the clinical relevance of notable toxicity findings, and the clinical context of the medicine’s use fosters effective dialogue and improves regulatory alignment. The WoE approach in ICH S11 ensures that relevant safety information is generated to support paediatric drug development while balancing the principles of non-clinical replacement, reduction and refinement (the 3Rs).

## Introduction

Generating robust evidence is essential to support the development of safe, effective, and high-quality medicines for the paediatric population. As children undergo extensive organ development and maturation, especially in the first years of life, the structural and functional differences in their organ systems can affect the pharmacodynamics (PD) and pharmacokinetics (PK) of medicines. This can result in underexposure and reduced efficacy, overexposure causing adverse effects, or unique sensitivities (1). To stimulate high-quality research into paediatric medicines, and to ensure that children have access to safe, effective, and age-appropriate medications, the European Union (EU) issued the Paediatric Regulation in 2007. This regulation made Paediatric Investigation Plans (PIP) compulsory for all new medicinal products (2). A PIP has to be agreed with by the Paediatric Committee (PDCO) at the European Medicines Agency (EMA) early in drug development and outlines quality, non-clinical and clinical plans (including timelines) to support the development and authorisation of a medicine for all relevant subsets of the paediatric population (3). The PIP may also include, when applicable, a justification to waive (part of) the paediatric population from the requirement to be included in paediatric studies (product-specific waiver). This waiver can be based on factors such as: the condition is not occurring in the paediatric population, the medicine is ineffective or unsafe for children, or the medicine does not offer a significant therapeutic benefit (2). Before submission of a Marketing Authorisation Application (MAA), compliance with all agreed study plans contained and completed in the PIP must also be checked by the EMA (2).

To support the safety evaluation of a novel medicine for paediatric use, specific non-clinical studies, including studies in juvenile animals, may be warranted. Over the years, different health authorities issued guidance defining the conditions to initiate Juvenile Animal Studies (JAS) (1, 3, 4). JAS studies were generally recommended when the preceding animal studies or human safety data were considered insufficient to support the safe use of a medicine in the paediatric population. While all guidelines emphasised that the need for a JAS should be evaluated on a case-by-case, science-driven basis, there was no consensus on when a JAS should be conducted. In addition, refined criteria to optimise study design were lacking (1, 5–7). This led to cases of large non-clinical juvenile study programs being conducted without a clear, scientific justification (8). JAS designs varied, even among compounds with similar therapeutic indications and paediatric age groups, and the relevance of findings was frequently compromised (9, 10). A specific guidance for the non-clinical development of medicinal products intended exclusively for paediatric use was also lacking (7, 11). To provide global guidance on the above issues, the ICH S11 guideline on Nonclinical Safety Testing in Support of Development of Paediatric Medicines was developed (12).

The main objectives of the ICH S11 guideline were to improve harmonisation between applicants and health authorities, to promote the timely conduct of paediatric clinical trials, to reduce the unnecessary use of juvenile animals, and to promote international standards for non-clinical testing of medicines intended for paediatric use (12).

The ICH S11 guideline was the first to require a standardised weight of evidence (WoE) approach to guide the nonclinical development strategy and determine the need for additional (juvenile) non-clinical studies. Importantly, the WoE considerations integrate clinical information (e.g., youngest intended patient age, the amount/type of existing data, clinical treatment duration), pharmacological properties (e.g., pharmacology-related or non-clinical effects on developing organs, the role of the pharmacological target in development, and drug specificity), and PK data. Where relevant, additional factors are recommended for consideration, such as clinical risk mitigation strategies or the feasibility of performing a study in the selected species. If a JAS is deemed necessary, the guideline provides suggestions on how to customise the study design to best support the paediatric development. Notably, the guideline introduces the concept of core endpoints, which should always be included, and additional endpoints, driven by specific identified concerns. These harmonised WoE considerations were expected to provide a scientific basis for communication and discussion on questions concerning JAS during regulatory interactions (12).

**Figure 1:**
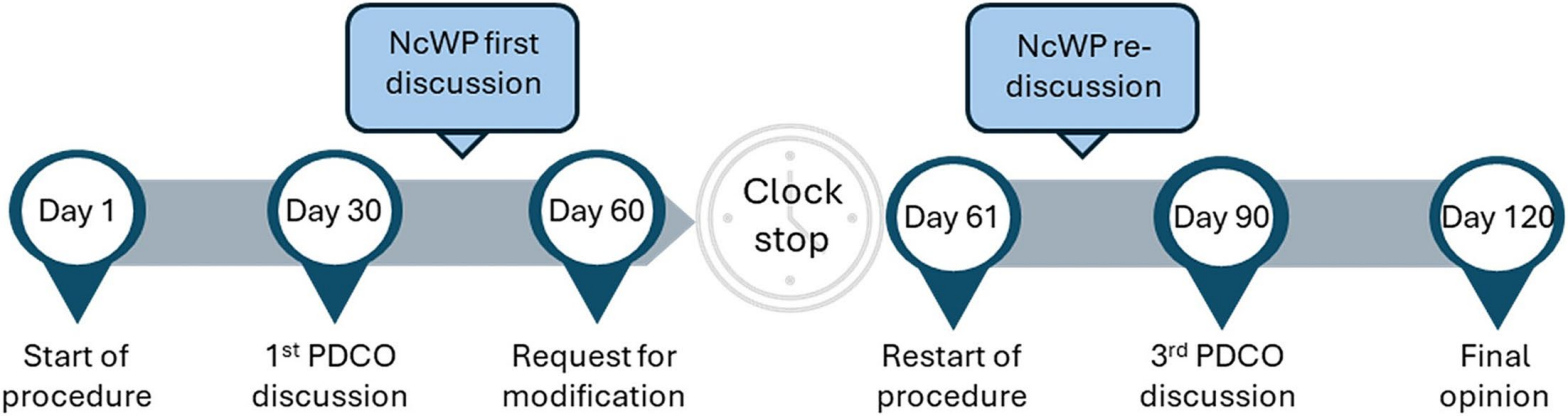
PIP lifecycle. Following the submission of a PIP an assessment process of 120 days is started, with 4 PDCO meetings taking place on days 30, 60, 90 and 120. After day 30, if requested by the PDCO, the NcWP reviews in-depth the non-clinical package of a PIP and can request additional information from the applicant. At day 60 the requests for modification are sent to the applicant and the PIP goes into clock-stop until the applicant responses are submitted. Once these are submitted the procedure restarts and the answers are reviewed by the assessment team and NcWP, followed by re-discussion at the day 90 PDCO meeting. If further doubts remain, questions can be sent to the applicant for response before day 120 conclusion and final opinion adoption. Non-clinical Working Party (NcWP), Paediatric committee (PDCO), Paediatric Investigation Plan (PIP).

As a result, non-clinical WoE approaches to identify the need for and design of a JAS have been integrated into the PIP application and are assessed by the PDCO, supported by the EMA Non-clinical Working Party (NcWP). During the PIP review process, requests for clarification or modification to the proposed non-clinical program can be made (Figure 1).

Here, we aim to evaluate, for the first time since the implementation of the ICH S11 guideline in September 2020 in the EU, how its introduction shaped the non-clinical development programs for paediatric medicines. This retrospective review of JAS proposals in PIPs analyses how ICH S11 was implemented by pharmaceutical companies and EMA, and whether the objectives of the guideline were met. The aim of this manuscript is to provide insights into the agreement between applicants’ proposal and EMA assessment (further referred to as regulatory alignment) and offer recommendations to improve future discussions around the need for and design of a JAS.

## Methods

We performed a retrospective analysis of assessed and agreed PIPs by the PDCO between November 2020 and October 2023.

For each PIP, we gathered the following documents from the EMA database: EMA/PDCO Summary Report, the non-clinical assessment form from the PDCO Non-clinical Working Group (until 2022) or NcWP (2022 onwards, both further referenced as NcWP), and the minutes of the NcWP and PDCO discussions. These documents were used to collect information on the product and to identify the applicants’ and EMA positions regarding the conduct of a JAS based on the WoE approach.

The PDCO assesses all PIPs and refers a selection of applications to the NcWP when an in-depth non-clinical WoE review is required. PIPs that were not referred to the NcWP were excluded from the analysis. The number of PIPs included was further refined with the following exclusion criteria: PIPs in clock stop; PIPs that were referred to the NcWP with questions unrelated to JAS; full waiver applications; applications that were withdrawn before a final PDCO decision was reached; PIP modifications; applications where JAS plans were previously discussed by NcWP on preceding PIPs and products out of the scope of the ICH S11 guideline (vaccines, gene and cellular therapies, and tissue engineered products).

We created a database that was divided into six main sections: (1) basic product information, (2) the available non-clinical data at the time of submitting the PIP, (3) the applicant’s position on whether a JAS was considered needed and the related justifications, (4) the NcWP assessment of the applicant’s proposal, (5) requests for clarification by EMA to the applicant if applicable, (6) the final conclusion on the need for and design of a JAS based on the PDCO opinion. The basic product information included procedure number, the date of PIP submission, the timing of PIP according to the Paediatric Regulation, International Non-proprietary Name (INN), active substance, modality type, therapeutic area, target and mechanism of action of the product, intended adult indication, intended paediatric indication, the target patient age proposed by the applicant, the final patient age agreed by the PDCO and the treatment duration. In order to collect information on the non-clinical package, the completion status of studies related to repeated dose toxicity (RDT), developmental and reproductive toxicity (DART), carcinogenicity, and genotoxicity was recorded. Finally, a qualitative analysis of the following metrics was performed: the applicant’s WoE, NcWP assessment, whether the NcWP agreed on the provided WoE and the underlying reasons, the aspects that were considered lacking in the applicant’s WoE discussion according to the NcWP and the applicant’s response, and the final EMA conclusion. These parameters were coded for the purpose of data aggregation and analysis. The number of applications with JAS removed, modified, added, or unchanged was analysed. For cases where the applicant received requests to revise the JAS design, the changes in the study design were examined.

## Results

### Data inclusion/exclusion

**Figure 2:**
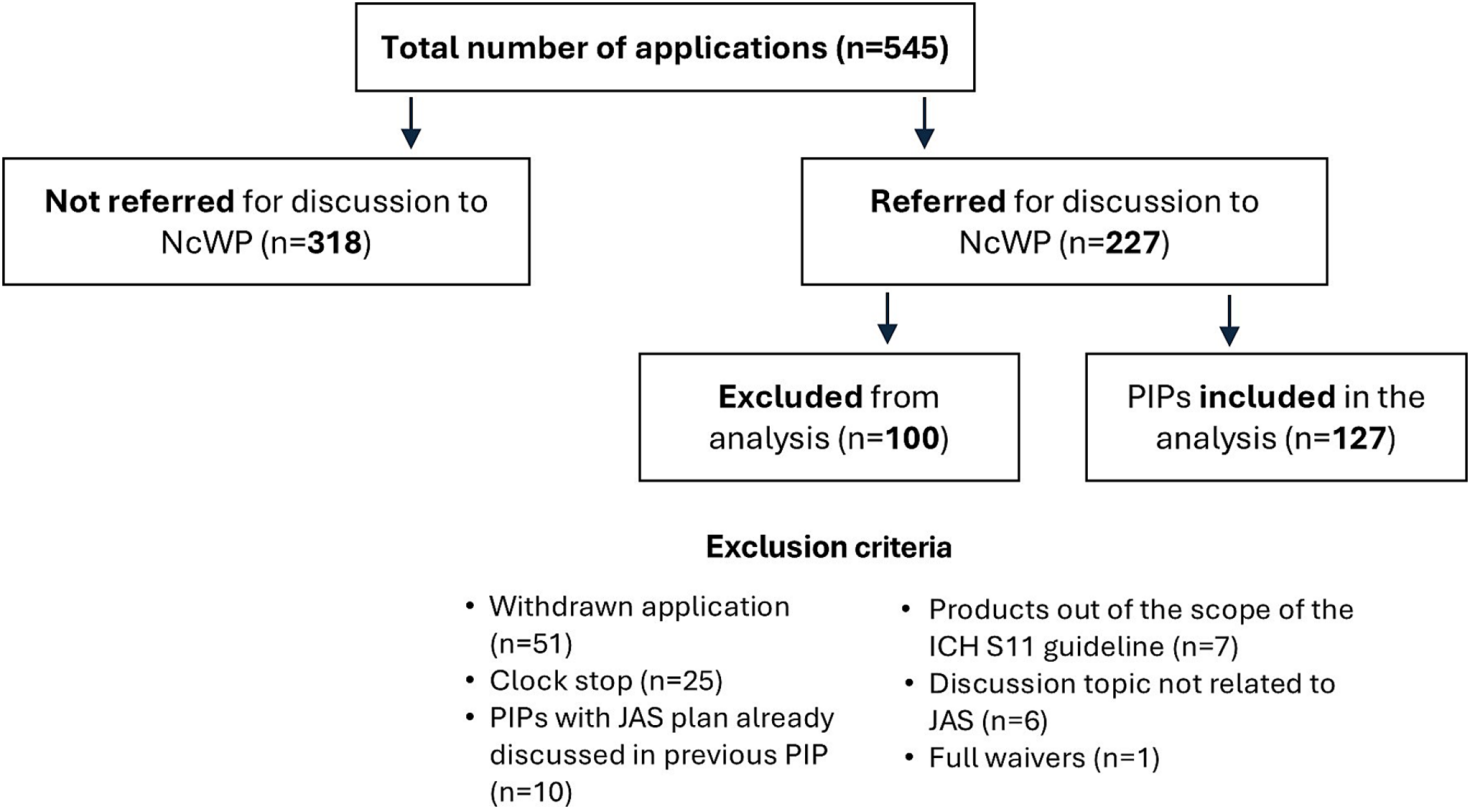
Data inclusion and exclusion in the analysis. International Council for Harmonisation (ICH), Juvenile Animal Studies (JAS), Non-clinical Working Party (NcWP), Paediatric Investigation Plan (PIP).

In total, we gathered 545 PIPs approved between November 2020 and October 2023 from the EMA database. Of these, 318 were excluded, as they were not referred to the NcWP for additional non-clinical review. Of the 227 PIPs discussed by the NcWP, 100 PIPs were further excluded because they were either withdrawn applications (*n* = 51), applications in clock stop (*n* = 25), PIPs where the proposed JAS had already been discussed in previous application(s) (*n* = 10), products out of the scope of the ICH S11 guideline (*n* = 7), topics not related to the need for or design of a JAS (*n* = 6), or PIPs where full waivers applied (*n* = 1). The remaining 127 applications were included in the analysis (Figure 2).

### Overview of the dataset

**Figure 3:**
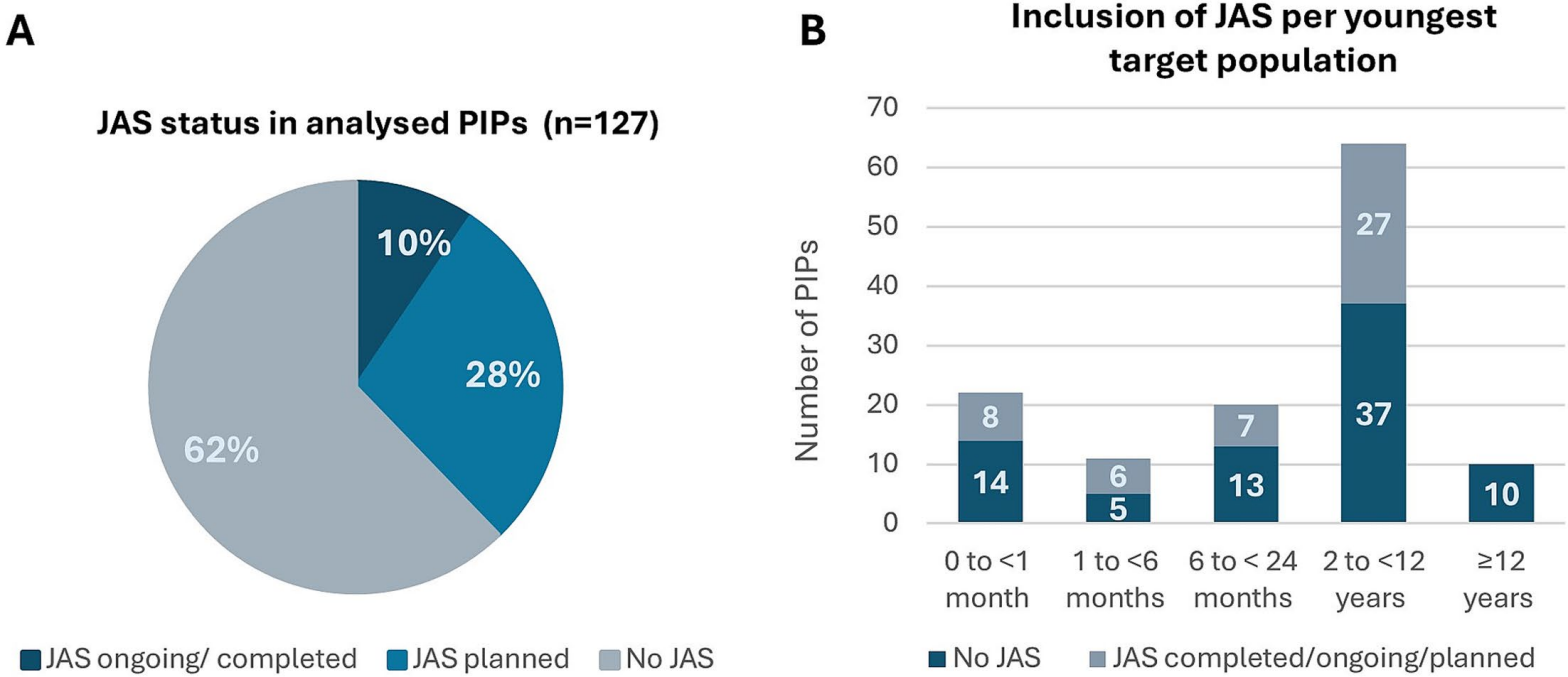
Overview of the PIPs included in the analysis (*n* = 127): applicant’s proposal on Day 0. **(A)** PIPs distribution regarding the status of JAS – not planned, planned or ongoing/completed. **(B)** Age distribution of the youngest target population in the PIPs and its association with JAS. Juvenile Animal Studies (JAS), Paediatric Investigation Plan (PIP).

Among the 127 PIP applications that met the project’s inclusion criteria, no JAS were planned in 62% (79/127) cases. In 28% (36/127) of the cases JAS were planned, while in 10% (12/127) of the cases JAS were already ongoing or completed at time of the initial submission (Figure 3A). Forty-nine JAS were included in 48 applications. Juvenile rats were the predominant rodent test species (37/49, 76%) followed by mice (3/49, 6%). Non-rodent JAS involved dogs (3/49, 6%), non-human primates (NHP) and mini pigs (both 2/49, 4%). One application included JAS in both mice and in NHP. Juvenile NHP studies refer to studies in monkeys aged 10–14 months at study initiation. Studies in older monkeys were not counted, as these were part of the standard toxicity package and not dedicated JAS. It is acknowledged though that cynomolgus monkeys used for general toxicity testing are frequently within the 2- to 3-year age range, representing a peripubertal age for females and a prepubertal age for males.

When stratifying the PIPs referred to the NcWP into age categories, based on the youngest intended patient age as proposed by the applicants, the distribution was as follows: patients of <1 month old (22/127, 17%), 1 to 6 months (11/127, 9%), 6 to <24 months old (20/127, 16%), 2 to <12 years old (64/127, 50%), and ≥ 12 years old (10/127, 8%) (Figure 3B). In applications where the youngest intended populations were below 12 years old (*n* = 117), 69/117 (59%) had no JAS planned, while 48/117 (41%) had JAS planned, completed, or ongoing. When the youngest intended population was 12 years of age or older (*n* = 10), no dedicated JAS were planned (Figure 3B). Of note, the age distribution in the analysis is affected by the inclusion criteria, as a large portion of PIPs with a development in adolescent patients only were not referred to the NcWP for assessment and were therefore excluded from the current analysis.

The products in the 127 applications were distributed across 11 Anatomical Therapeutic Chemical (ATC) Classification level 1 categories. Antineoplastic and immunomodulating agents (L) (27/127, 21%), alimentary tract and metabolism (A) (23/127, 18%), and anti-infective for systemic use (J) (17/127, 13%) represented more than 50% of the applications (Supplementary Figure S1). Regarding the presence of JAS in the plans proposed by the applicant, 70% or more of the applications in both nervous system (N) and muscular-skeletal system (M) included JAS. In contrast, only 15% of the proposed PIPs for antineoplastic and immunomodulating agents (L) included a JAS that was planned, ongoing or completed (Supplementary Figure S1).

### Absence of JAS in the PIPs

**Figure 4:**
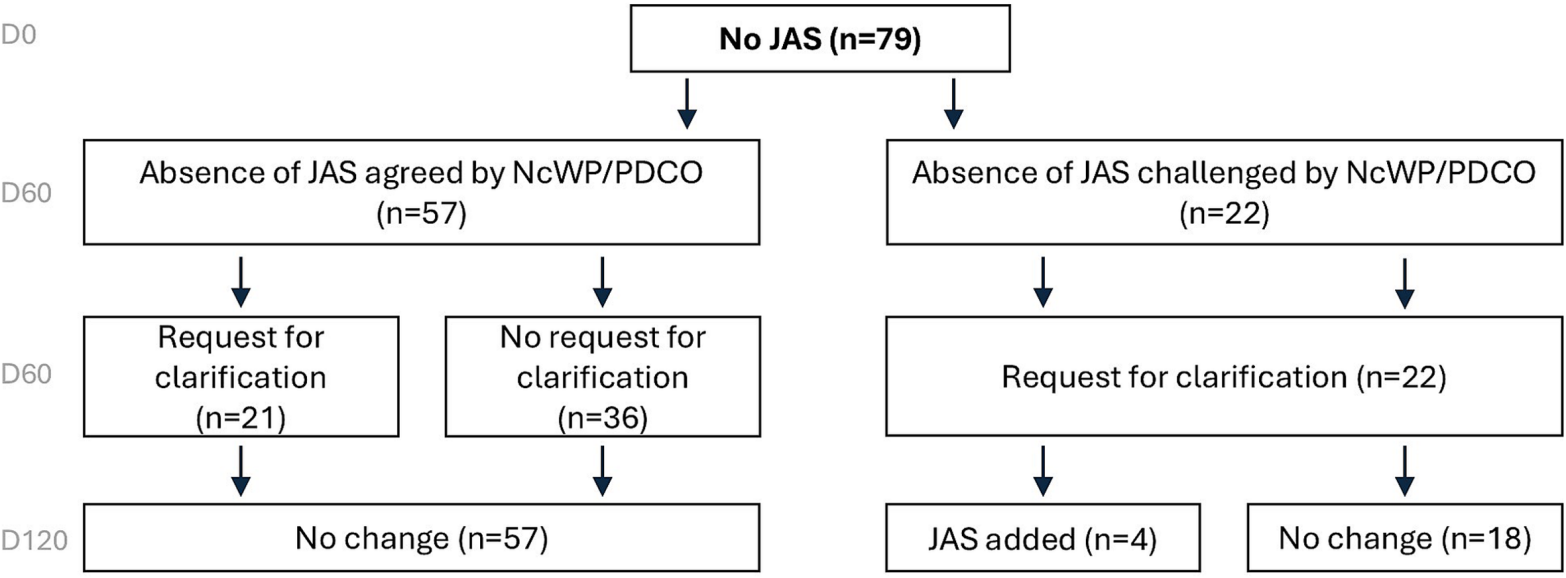
Overview of JAS decisions based on EMA assessment on Day 120 for PIPs with no JAS proposed. When not originally planned, JAS were rarely added to PIPs. European Medicines Agency (EMA), Juvenile Animal Studies (JAS), Paediatric Investigation Plan (PIP).

Of the 79 applications in which no JAS were proposed, 57 were agreed and 22 were challenged by EMA on Day 60. Of those 22 applications, 4 had a JAS added to the opinion on Day 120 (Figure 4).

#### Applications where the absence of JAS was agreed (*n* = 57)

In 57 applications, the absence of a dedicated JAS was agreed by EMA. In 36 of these 57 applications (63%), this absence was accepted without any further questions. Overall, data on RDT (52/57, 91%), DART (20/57, 35%), off-target screening (10/57, 18%), safety pharmacology (2/57, 4%), and/or clinical data (37/57, 65%) were used to support the absence of a JAS (Table 1). However, in 21/57 (37%) applications, information regarding the completed non-clinical safety studies or potential pharmacology-related risks on developing organs was missing. As a result, the EMA sent requests for clarification (more than one request can apply per case). The applicants were primarily asked to elaborate on the clinical relevance of specific non-clinical findings, address potential pharmacology-mediated effects on developing organs, provide additional details on completed non-clinical studies, or reconsider the addition of a JAS when further data becomes available (Table 1).

**Table 1:** Factors supporting the absence of a JAS and requests for clarification (*n* = 57).

*FINAL* youngest intended patients		< 1 month (*n* = 13)	1 month - < 6 month (*n* = 6)	6 month - < 24 month (*n* = 13)	2 year - < 12 year (*n* = 23)	≥ 12 year (*n* = 2)	Total (*n* = 57)
Were requests for clarification partly due to waiver changed? (Day 60)	Yes	4	2	5	8	0	19
	No	9	4	8	15	2	38
Factors considered by the applicant (day 60)	**Non-clinical data**						
	Repeated-dose toxicity	12	4	13	21	2	52
	Young/adolescent animals^1^	3	1	8	14	–	26
	Juvenile animals^2^	1	–	1	1	–	3
	FEED/EFD/(e)PPND	4	2	3	11	–	20
	Off-target investigations	3	3	1	2	1	10
	Safety pharmacology	1	–	1	–	–	2
	**Clinical data**						
	Adult	10	4	8	12	2	36
	Paediatric	–	1	–	–	–	1
	**Pharmacological properties**						
	Discussion on PD-related effects or effects on developing organs	9	2	11	14	1	37
	High selectivity for target	2	2	4	3	1	12
	**Feasibility**						
	JAS is not feasible	2	2	2	7	–	13
	Other	11	6	14	9	0	40
Requests for clarification (day 60)	Discuss the relevance of presence or absence of non-clinical findings and/or theoretical PD-related effects on developing organs	3	3	–	10	1	17
	**Provide additional data**						
	(e)PPND results	–	1	1	2	–	4
	Target expression	1	–	1	–	–	2
	Exposure margins	1	–	1	–	–	2
	Age of animals used in RDT	–	–	–	2	–	2
	Method: off-target effect	1	–	–	–	–	1
	Iterative approach: re-consider a JAS when more data become available	–	–	1	1	–	2

^1^ = 4–9 week-old rats, 6–9 week-old mice, or 2–7 year-old monkeys; ^2^ = rats of PND 21–23 (e) PPND = enhanced PPND.

In addition, other factors supporting the absence of JAS were part of the overall WoE discussion provided by the applicants. These arguments included lack of study feasibility due to the inability to achieve clinically relevant exposures, the unavailability of relevant juvenile models or the technical feasibility; the known literature-reported paediatric risks on same-in-class compounds; the clinical manageability of identified risks; benefit/risk considerations; clinical safety monitoring considered as satisfactory; short treatment duration; and global regulatory advice (Supplementary Table S1). Overall, for all of these applications (n = 57) the EMA agreed that the WoE analysis indicated that additional investigations in JAS were not needed.

### Applications where the absence of a JAS was challenged (*n* = 22)

For 22/79 (28%) applications the absence of a JAS was challenged by EMA. In 10/22 (45%) applications, the PDCO refused the initially proposed waiver, which contributed to requests for clarification and modification that were sent to the applicants (Table 2). These requests primarily focused on the results of completed non-clinical safety studies and intended to provide additional discussion on the clinical relevance of, or mechanisms of action behind observed toxicities. Additional requests related to pharmacological properties (e.g., role of the pharmaceutical target in organ development) and other aspects, such as clarifying the clinical monitoring plan for certain endpoints, determining whether literature data on similar molecules were available to assist risk characterisation, and exploring whether changes in the dosing regimen could increase the feasibility of a JAS (Table 2). The questions regarding non-clinical data were mainly to address potential concern for central nervous system (CNS) or reproductive organs toxicity (Supplementary Table S2). Issues were resolved in 18/22 (82%) applications. In 4 cases, a JAS proposal was added to the PIP, after an updated WoE was submitted (see case examples in Box 1).

**Table 2:** Requests for clarification on day 60: the absence of a JAS was challenged (*n* = 22).

*FINAL* youngest intended patients		< 1 month (*n* = 8)	1 month - < 6 month (*n* = 1)	6 month - < 24 month (*n* = 5)	2 year - < 12 year (*n* = 6)	≥ 12 year (*n* = 2)	Total (*n* = 22)
Was the absence of a JAS challenged partly due to a changed waiver? (day 60)	Yes	2	1	3	2	2	10
	No	6	0	2	4	0	12
Requests on non-clinical data (day 60)	Clinical relevance (absence) of effects on developing organs	4	1	4	12	2	23
	MoA of non-clinical findings	3	–	1	3	–	7
	Results/relevance of PPND study	1	1	1	–	–	3
	Data on off-target effects	2	–	–	1	–	3
Requests on pharmacological properties (day 60)	MoA of compound	2	–	2	–	–	4
	Role of the target/PD in development	4	1	1	–	–	6
Other requests (day 60)	Clinical monitoring plan	2	1	–	–	–	3
	Literature data with similar drugs	1	–	–	1	2	3
	Relevance of data with surrogate molecule	1	–	–	–	–	1
	Predictivity of species	1	–	1	–	–	2
	Receptor specificity vs. other compound in class	–	–	–	–	1	1
	Feasibility of JAS	1	–	–	–	–	1
NcWP/PDCO conclusion on Day 120	No change	7	1	4	4	2	18
	JAS added	1	0	1	2	0	4

BOX 1 **Case examples of rationales for adding a JAS in the PIP.**

**CASE 1**

**Table:** 

**Proposed paediatric population:** 2yr - < 18yr	**Agreed population:** 2yr - < 18yr
**Non-clinical safety programme:** Completed safety pharmacology in rats and dogs, toxicity studies up to 16 weeks in rats and dogs, EFD in rats and rabbits, genotoxicity studies in vitro and in rats. Chronic RDT in rats and dogs, FEED and PPND in rats were planned.	**Target organs of toxicity:** CNS, liver, adrenal glands, immune system, gastrointestinal (GI) tract

**Applicant’s position:** The target for this compound was known to have a role in the regulation of the immune system and in epithelial survival in the intestine. The adverse effects observed in the immune system and GI tract were considered to be monitorable in the clinic and not to be a specific concern for paediatric patients ≥ 2 years old. Sedation-like effects and anticonvulsant activity were observed in the safety pharmacology study in rats. The 2-week non-GLP study in rats showed convulsions (in one animal) and respiratory alteration at the high dose. At lower doses, reduced activity, gait changes, and/or decreased muscle tone were observed. The GLP dog studies revealed convulsions (in one animal) and ataxia at the high dose, and tremors at lower doses. In the 16-week dog study, adverse liver effects at the higher dose were also observed. Minimal to moderate adrenocortical hypertrophy and/or vacuolation was observed in the 4-week and 16-week rat study. The applicant was of the opinion that these findings were of limited clinical relevance as phase I clinical data did not reveal severe adverse events that were related to pharmacology or potential off-target effects. The absence of a JAS to evaluate effects on the CNS, liver, immune system, and adrenal gland, was justified by the argument that these organs are not in a critical period of development in patients aged 2 years and older.

**NcWP:** The clinical risk profile of the compound remained inadequately characterised due to the limited data derived from a small number of participants enrolled in phase I clinical trials. Adverse CNS effects were noted in rats and dogs at clinically relevant exposure levels. No discussion was provided regarding the potential mechanisms underlying these CNS effects, the distribution of the compound to the CNS, or whether the target is expressed in the brain. Given these uncertainties and the continuous development of the CNS until adulthood, the applicant was requested to propose a JAS to address potential concerns related to CNS development. The necessity for the JAS could be reassessed based on new data, such as that from the planned chronic RDT studies.

**Updated WoE:** A JAS with additional neurobehavioral assessments (motor activity, acoustic startle, water maze) was planned to address the potential concerns for CNS development. The applicant was recommended to reassess the necessity for the JAS study upon completion of chronic RDT or other studies.

**CASE 2**

**Table:** 

**Proposed paediatric population:** 6yr - < 18yr	**Agreed population:** 1yr - < 18yr
**Non-clinical safety program:** Completed safety pharmacology in rats and mini pigs, toxicity studies up to 39 weeks in rats and mini pigs, genotoxicity studies in vitro and in rats, FEED in rats, EFD in rats and rabbits, PPND in rats. Carcinogenicity study in rats planned.	**Target organs of toxicity:** Reproductive organs

**Applicant’s position:** Chronic RDT in adolescent animals (8-week rats and 2.5- to 4.5-month mini pigs) did not suggest overt toxicity up to exposure margins of 14-62x for female and male rats, respectively, and ~19x for mini pigs compared to the expected clinical exposure. Above these exposures, main findings were mortality in the highest doses tested in both species and below those, decreased body weight and food consumption in rats. There was no indication of organ specific toxicity. Toxicity studies in juvenile animals were not considered necessary to support the administration of the compound to adolescents in Phase III clinical trials. The dose selection for the PK/PD clinical study in patients aged 6 to 11 years would be based on the data derived from adults and adolescents.

**NcWP:** In this late submission, conventional RDT studies in pubertal rats and mini pigs did not identify target organs of development, with the exception of the epididymides, which showed decreased weight in mini pigs and rat pups in the PPND study. There was no histopathology data to further evaluate this finding. Decreases in body weight and food consumption were observed in rats but not in the mini pigs RDT or DART studies. Because the mode of action and target for this compound were not fully elucidated, it was challenging to identify an appropriate species in terms of PD. Data in rats did not suggest a pharmacological response whereas the mini pig data did. Without additional pharmacological data, a complete safety profile could not be generated and it was uncertain whether the absence of target organ toxicity was due to testing in a non- or less relevant species. The lowering of the waiver cut-off to 1 year of age also required additional discussion. The applicant was requested to update the WoE discussion on the need for a JAS taking into account the younger target population.

**Updated WoE:** The compound was reported to cross the blood-brain barrier (BBB) without corresponding clinical evidence of CNS effects, nor did a broad pharmacologic screen reveal binding to known receptors in the CNS. Thus, the observed weight loss was believed to be a systemic effect. A more in-depth discussion of the target and the relevance of species was not provided. The applicant proposed a JAS in rats starting at PND 7 to support the administration of the compound to patients from 1 year onwards. The justification for the species selection was based on the metabolic profile similarity between rats and humans. However, NcWP did not consider the rat to be a relevant model, except for evaluating off-target effects. Nevertheless, the rat JAS was provisionally accepted to evaluate off-target effects on CNS and spermatogenesis. The study design was extensively modified by NcWP, including delaying the starting age to PND 14 to better match children from 1 year onwards. In addition, a measure was included to generate further PD data to define the molecular target and mode of action. The applicant was recommended to reassess the necessity or design of the JAS once this new data was generated.

Abbreviations: NOAEL (No-Observed-Adverse-Effect Level); PPND (Pre- and Postnatal Development); FEED (Fertility and early Embryonic Development); EFD (Embryo-Fetal Development); PND (post-natal day).

### JAS planned in the PIPs

**Figure 5:**
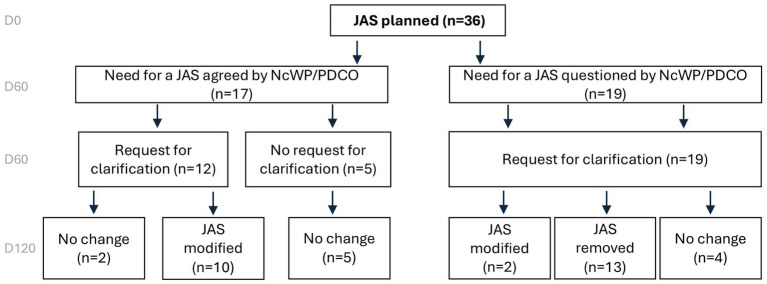
Overview of JAS decisions based on EMA assessment on Day 120 for PIPs involving a planned JAS. When planned, JAS were removed in about a third of the cases. European Medicines Agency (EMA), Juvenile Animal Studies (JAS), Paediatric Investigation Plan (PIP), Non-clinical Working Party (NcWP).

Of the 127 applications included in this analysis, a JAS was planned in 36 cases. In 17/36 (47%) applications the need for a JAS was agreed and in 19/36 (53%) cases the need for a JAS was challenged by EMA (Figure 5).

### The need for a JAS was agreed (*n* = 17)

Out of 17 applications, paediatric risks were identified by the applicant in 13 applications (76%) based on non-clinical, clinical, and/or pharmacology-related effects on developing organs (Table 3). The applicants proposed a JAS primarily to characterise potential adverse effects on the CNS (*n* = 5), reproductive organs (*n* = 3), bone marrow (*n* = 3), and gastrointestinal tract (n = 3) and a range of other target organs (Supplementary Table S3). However, in 4/17 (24%) applications, no clear WoE discussion or scientific rationale was provided by the applicant to support the need for a JAS. In two of these applications a JAS was included following requests of other health authorities.

**Table 3:** Factors supporting the need for a JAS considered by the applicant (*n* = 17).

*FINAL* youngest intended patients		< 1 month (*n* = 3)	1 month - < 6 month (*n* = 2)	6 month - < 24 month (*n* = 2)	2 year - < 12 year (*n* = 10)	Total (*n* = 17)
Was the waiver changed? (day 60)	Yes	1	1	1	4	7
	No	2	1	1	6	10
Factors related to available data with the molecules and PD	**Non-clinical data**					
	Repeated-dose toxicity	3	1	1	8	13
	Young/adolescent animals	–	–	–	4	4
	FEED/EFD/(e)PPND	–	–	–	4	4
	Off-target investigation	1	–	–	1	2
	Safety pharmacology	1	–	–	3	4
	**Clinical data**					
	Adult	2	–	–	–	2
	Adolescent	1	–	–	–	1
	**Pharmacological properties**					
	Discussion on PD-related effects or effects on developing organs	2	1	1	10	14
Other factors	No factor identified/unclear rationale	1	1	1	1	4
	Regulatory advice	1	–	–	2	3
	Literature data with similar drugs	–	–	–	2	2
	Effects cannot be monitored in clinic	–	–	–	1	1
The added value of a JAS considered by the applicant	JAS was considered required to characterise adverse effects on developing organs	5	3	8	14	30
	JAS was considered required to define exposure margin	–	–	–	1	1

Of the 17 applications, 5 (29%) had no requests for changes to the original proposed plans made by EMA. The remaining 12 applications received requests for clarification to provide additional information on the toxicity findings, to justify the proposed animal species (e.g., justify the use of dogs instead of rodents), or to amend the design of the JAS (e.g., age of the animals at study start, dosing period, addition of endpoints). After the clock-stop, in 10 out of the 12 cases the JAS design was modified (for modifications to the JAS design, see the section below and Table 4). In the remaining 2 cases, no changes were made to the JAS study design. In one case the study design remained the same to accommodate the requests of another health authority. In the other case, the JAS was already ongoing when the requests for modification were sent to the applicant, making it unfeasible to adopt the suggested changes to the study design.

**Table 4:** Modifications to the JAS design in the final opinion (*n* = 12).

*FINAL* youngest intended patients		< 1 month (*n* = 2)	1 month - < 6 month (*n* = 2)	6 month - < 24 month (*n* = 1)	2 year - < 12 year (*n* = 7)	Total (*n* = 12)
Modifications to the youngest starting age of animals (rat only)	**Age of animals increased**					
	PND4/7 → PND10/14	–	2	1	1	4
	**Age of animals decreased**					
	PND10 → PND7	1	–	–	–	1
	PND28 → PND21	–	–	–	1	1
Modifications to the endpoints	**Endpoints removed**					
	CNS assessment	1	–	–	1	2
	Ophthalmologic exams	1	–	–	1	2
	Bone density assessment	–	1	–	–	1
	Reproductive assessment	1	–	–	–	1
	**Endpoints added**					
	CNS assessment	1	2	–	3	6
	Bone histology, density, biomarker for formation and resorption	1	–	–	1	2
Modifications to the dosing or recovery period	Dosing duration and recovery period extended	–	–	–	3	3
Modifications to the timing of CNS measurement and dose group		–	–	–	4	4

### The need for a JAS was disagreed or questioned (*n* = 19)

In 19/36 (53%) cases the need for a dedicated JAS was challenged by EMA. Requests for clarification were made regarding the scientific rationale behind the planned JAS, non-clinical data, target expression, and suggestions for the JAS design (Figure 5).

In 13 of these 19 applications, the planned JAS was removed from the PIP in the final opinion. According to EMA, the WoE evaluation did not support the need for additional non-clinical investigations in these cases. Arguments for this disagreement with the applicant’s initial position included the absence of identified target organs of toxicity (5/13, 38%), the fact that the identified target organs were not undergoing critical structural and functional development in the intended population (5/13, 38%), and the limited clinical relevance of the identified toxicity (3/13, 23%, Table 5). Additionally, other factors supporting EMA’s decision not to warrant a JAS included: when adverse effects of concern were clinically manageable, the safety profile was known based on literature with similar molecules, or the treatment duration was short (Table 5).

**Table 5:** Considerations leading to the removal of JAS and requests for clarification.

*FINAL* youngest intended patients		< 1 month (*n* = 1)	1 month - < 6 month (*n* = 1)	6 month - < 24 month (*n* = 1)	2 year - < 12 year (*n* = 10)	Total (*n* = 13)
Was the planned JAS challenged partly due to a changed waiver? (Day 60)	Yes	0	0	1	1	2
	No	1	1	0	9	11	Factors supporting the absence of a JAS considered by EMA
No target organs identified	1	1	–	3	5
Target organs not undergoing critical structural and functional development	–	–	1	4	5
Limited paediatric clinical relevance of the identified toxicity	–	–	–	3	3
Target has no role in organ development	–	–	–	1	1
Lack of feasibility of JAS	–	–	–	1	1
Effects of concern clinically manageable	–	–	1	2	3
Safety profile known with similar molecules	–	–	–	2	2
Stepwise approach in clinic	–	–	–	1	1
Short treatment duration	1	–	–	–	1

In 6 of these 19 applications, the proposed JAS remained part of the PIP. In 4 cases, no changes were made to the JAS plans following the requests for clarification. In the remaining 2 cases, the JAS design was modified.

Of the four cases with no changes to the proposal, two had the JAS initiated before day 60, making it unfeasible to adopt the EMA suggestions. In one case, the EMA agreed on the need for JAS and the proposed study design to address concerns regarding bone development after the applicant updated the WoE. In the remaining case, the JAS remained part of the PIP as the study was planned by the applicant in order to fulfil requests from other health authorities.

Of the two cases with a modified JAS design, one had the need for a JAS confirmed by the EMA to address liver toxicity after the WoE was updated. In the other case, the study endpoints were revised to optimise the design, and the JAS remained in the opinion despite the EMA’s WoE assessment indicating that it might not provide added value (for modifications to the JAS design, see the section below and Table 4).

### Modifications to JAS design (*n* = 12)

In 12/36 (33%) cases, the design of the planned JAS was modified (Table 4; Supplementary Table S4). To better align animal organ development to human organ development, the age of the animals at initiation of study was modified in 6 of these 12 cases (50%). This adjustment considered the youngest intended patient age and the developmental periods of organ(s) of concern. Additionally, in 5/12 (33%) cases, non-core endpoints were removed. These endpoints were deemed inadequate for addressing the observed concerns (e.g., functional observation battery (FOB) assessment not sensitive for CNS evaluation in a JAS), or there were no significant organ-specific concerns identified that would justify the inclusion of the additional endpoint (e.g., ophthalmologic assessments) after the updated WoE. Additional endpoints concerning neurohistopathology, detailed CNS clinical observations, or bone histology were included in 6/12 (50%) cases. The dosing or recovery period was extended for CNS and reproductive assessments in 3/12 (25%) cases. The remaining modifications concerned the addition of timepoints for CNS endpoints measurement (3/12, 25%).

### Waiver changes

**Figure 6:**
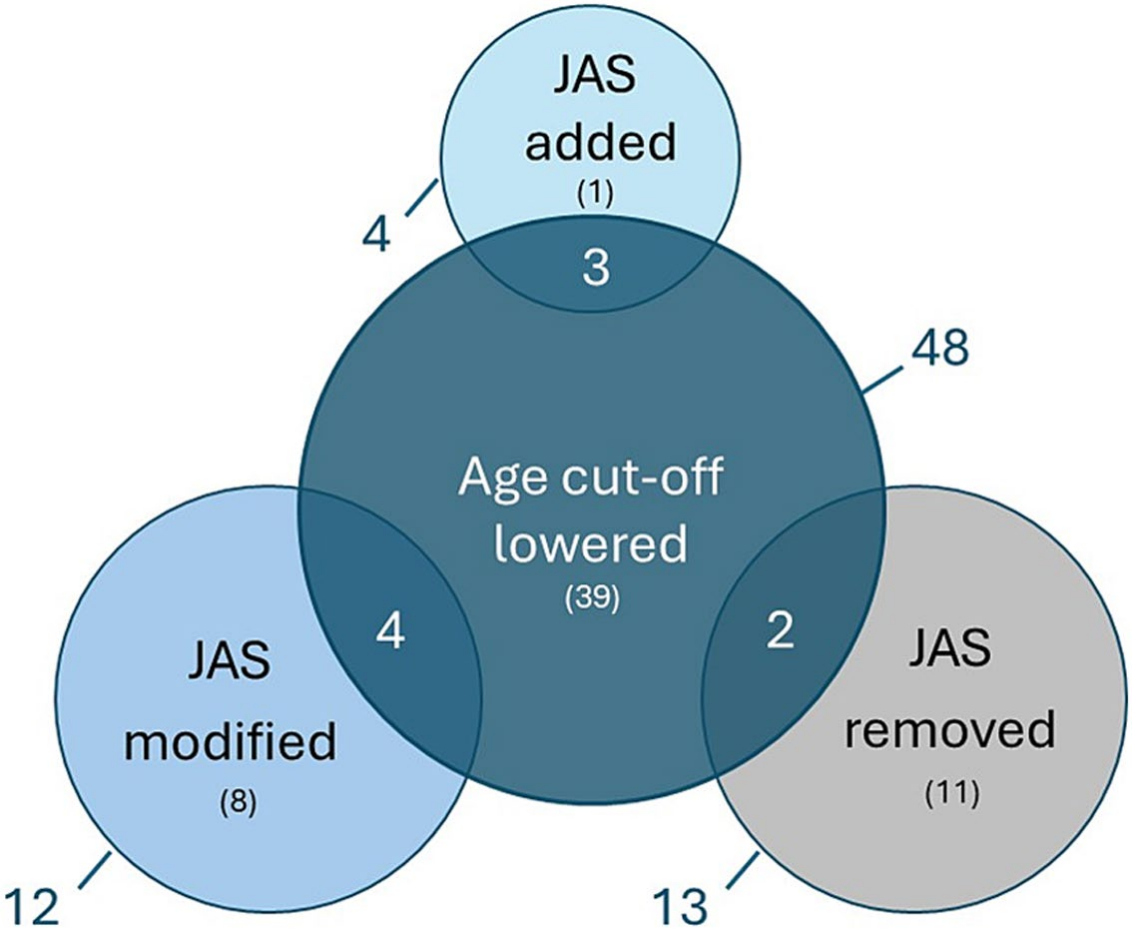
Overview of PIPs where the proposed waiver age cut-off was lowered versus the changes in the applicant’s initial JAS proposal. Nine out of 29 PIPs (31%) in which the JAS plan was changed had a lowered waiver age cut-off during the PIP assessment. The numbers outside of the circle represent the entire pool, the numbers in brackets represent non-overlapping subsets and the numbers in overlapping circles represent the overlapping pool. Juvenile Animal Studies (JAS), Paediatric Investigation Plan (PIP).

Of all the analysed PIPs, 48/127 (38%) had a lowered waiver age cut-off in the final opinion (Figure 6). In 39/48 (81%) of these cases, the change in the waiver cut-off did not affect the JAS plans for the PIP. In contrast, in 7/48 (15%) cases, the waiver changes contributed to modifications in the JAS plans or the addition of a JAS to the final PIP opinion. In 2/48 (4%) PIPs, the JAS was still removed despite the reduction in the waiver age cut-off. Overall, 9/29 PIPs (31%) in which the JAS plan was changed had a lowered waiver age cut-off during the PIP assessment.

### JAS completed or ongoing

Out of the 127 applications included in this analysis, 12 had a JAS already ongoing or completed. In 6/12 (50%) of the cases, requests for clarification were made to the applicants at day 60 discussion of the PIP evaluation (Supplementary Figure S2). These consisted of requests for additional information on clinical and non-clinical data or literature (*n* = 3), to discuss the non-clinical findings and their clinical relevance in the intended paediatric population (*n* = 3), for monitoring of adverse effects detected in juvenile animals in the clinical settings (*n* = 2), to further explore the potential for off-target effects via other non-clinical studies (*n* = 1), or to justify the proposed animal test species (*n* = 1). Of note, more than one request can be made to the applicants. None of these clarifications resulted in changes in the JAS itself, as the studies had already been initiated or completed.

## Discussion

The introduction of a globally harmonised WoE assessment in ICH S11 resulted in a science-based consideration of evaluating whether a JAS would be needed to support clinical trials in children of any age (12). We showed that when PIPs were submitted before JAS initiation, the scientific discussions based on the applicant’s WoE assessment resulted in agreement with the proposed non-clinical strategy in 75% of referred cases. This also applied to cases where the WoE supported paediatric clinical studies without a dedicated JAS (Figures 4, 5).

In about a quarter of cases, EMA disagreed with the WoE outcome and determined that changes in the non-clinical strategy were warranted to optimise the development plan for the intended patient population. This led to scientific discussions between applicants and EMA, resulting in the removal of 13 planned JAS (10%), modification of 12 planned JAS (9%) and the addition of 4 JAS (3%) to the opinion. Carleer and Karres conducted a similar analysis on the number of PIPs containing a JAS in the period between November 2008 and May 2010, prior to the implementation of ICH S11 (13). Of the 97 PIPs assessed, 33% initially included a JAS by the applicant, while an additional 26% had a JAS recommended by EMA. This indicates that following the implementation of ICH S11, the EMA adopted a more nuanced analytical approach regarding the addition of a JAS, in line with the 3R’s (Replace, Reduce, Refine) principles. However, this difference may not be solely attributed to the introduction of the guideline, as other factors may have influenced the significant decrease in requests by EMA. Over the last 15 years, advancements in understanding comparative organ maturation across species (14), along with the non-clinical community’s growing expertise in JAS designs and result interpretation, may also have contributed to this change.

In general, EMA disagreement with the applicant’s initial proposals was usually due to a WoE assessment that did not account for all risk factors identified in ICH S11 or lacked sufficient detail. It is not uncommon for WoE assessments, including those outside of juvenile toxicity, to require additional information to reach a decision if the WoE insufficiently discussed critical factors (15).

Even though applicants generally justified their non-clinical strategy based on the considerations recommended in the ICH S11 guideline (Tables 1, 3), requests for clarification were often sent by EMA (80/127; 63%). Discussions between the applicants and EMA focused on the clinical relevance of identified non-clinical findings for paediatric patients and possible pharmacology-related effects on developing organs (Tables 1, 2). Notable effects in conventional adult animal studies required explanatory discussions to understand the potential clinical implications in children. Ultimately, this in-depth discussion led to 64% (51/80) of the clarification requests not resulting in a change of the non-clinical strategy. This illustrates that a more careful consideration of developmental aspects of the products’ pharmacological target and providing sufficiently detailed discussions of notable toxicity findings would facilitate a more effective dialogue and regulatory alignment on the WoE outcome. We therefore advise to always consider the following factors to justify the clinical relevance of a finding or to argue for the potential need of a JAS: target organ maturity, distribution to potential target organs, mechanisms underlying observed adverse effects, animal-human (paediatric) exposure margins, age-related target expression, pharmacological effect on target organs, cross-species concordance, and contextual data from other non-clinical and clinical studies (Tables 1, 2).

For example, while the provided WoE for Case 1 (Box 1) was quite extensive, it lacked a thorough discussion on whether the identified CNS effects warranted a JAS for paediatric risk characterisation. CNS effects were observed in rats and dogs at relevant exposure levels. Because the potential mechanisms underlying these CNS effects, the distribution of the compound to the CNS, and whether the target is expressed in the brain were not sufficiently discussed throughout the procedure, a JAS was ultimately required.

Some less routinely used WoE factors, such as target selectivity and knowledge of the target, can impact the assessment of the need for a JAS. In Case 2 (Box 1), the absence of non-clinical findings in rats and minipigs was questioned due to the applicant’s insufficient discussion on the pharmacological relevance of the selected species. This resulted in a JAS being added. Furthermore, the WoE discussion can also include arguments less explicitly noted in ICH S11, such as the technical feasibility of a JAS, experience with other (similar) molecules, the ability to monitor or mitigate potentially clinically relevant findings in the clinic, or the intent to stagger clinical development in age cohorts to mitigate risk in younger patients.

The aim of an in-depth analysis of each non-clinical concern is to foster the design of fully informative JAS studies by considering a variety of factors (see Tables 1–5). In some cases, such analysis may conclude that a JAS study will not be informative or is unlikely to provide new insights. In many cases included in this review, a WoE exercise helped the applicant determine that a JAS was not valuable for characterising safety concerns or was not feasible to conduct (n = 75). For an additional 13 PIP applications, after an updated WoE assessment following interaction with EMA, JAS studies were removed from the PIP (Table 5). Notably, most cases where the removal of a JAS was possible involved developments targeting paediatric patients over 2 years of age (n = 10). This was partly due to a lack of concern for actively maturing and developing organs. When toxicologically relevant target organs have reached structural and functional maturity in the target paediatric population, it is generally accepted that a JAS, in addition to the standard non-clinical programme, is not necessary, as the risk can be adequately characterised in older (mature) animals. In these cases, depending on the target organ of concern, an identified risk may be more effectively characterised in the clinical (paediatric) program through dedicated safety monitoring endpoints.

For products targeting a youngest paediatric population between birth and 2 years of age, substantially fewer cases were noted where a proposed JAS was removed (n = 3). Despite the young age of the target population, the JAS could sometimes still be removed from the application in light of lack of age-specific target organs of concern and the very short treatment duration mitigating the risk. Thus, the regulator identified additional opportunities to reduce unnecessary testing in animals, thanks to a careful analysis of all elements in the WoE (Table 5). Taken together, our data demonstrates that a detailed WoE discussion helps to assess the added value of a JAS and gives an opportunity to design a more efficient development strategy.

### Innovative aspects of the guideline

Three considerations that underscore the importance of an early consideration of the non-clinical strategy for paediatric pharmaceutical development as recommended in ICH S11 were identified.

First, before an appropriate non-clinical plan can be developed, it is essential to understand the paediatric clinical development plan. The ICH S11 guideline placed greater emphasis on integrating the clinical context and clinical safety information into the WoE discussion. Table 1 illustrates that such factors were actively considered to support the absence of JAS in PIPs. For example, a summary of the clinical safety data with respect to the identified concerns is often provided to discuss the clinical relevance, or lack thereof, of certain non-clinical findings. Additionally, arguments relating to the clinical context were integrated in the WoE discussion, such as staggered clinical approach, monitorability and manageability of the risks in the clinical setting, benefit/risk considerations and treatment duration. Similarly, clinical considerations contributed to the removal of JAS by EMA (Table 5). On the other hand, in at least one case, the inability to adequately monitor the concerning effects in the clinical setting was included as a reason to support the need for a JAS (Table 3).

Secondly, specific considerations for paediatric-first/only development were introduced in the ICH S11 guideline. Our data set included 5 products, which were developed specifically for paediatric-only indications. All of these included JAS in their non-clinical package to support the paediatric-only indication. In one of these PIPs, JAS in two species (NHP 14 months old and mice PND 7 at study start) were ongoing to support an early phase clinical trial in paediatric patients from birth. For this limited number of cases, the principles outlined in ICH S11 seemed to have been followed, although any additional impact of the guideline on this matter cannot be further analysed based on our data set.

Finally, another recommendation in ICH S11, was to encourage early non-clinical support in the development of paediatric pharmaceuticals. One way to timely address potential safety concerns for paediatric patients is to modify the design and/or timing of the standard non-clinical program. As shown in Table 1, in 26 cases, the ‘standard’ RDT studies within the non-clinical program were initiated in young and/or adolescent animals, including, for example, 2-year-old monkeys or 3 to 4-week-old rats. Another approach could involve conducting the PPND study earlier than usual, incorporating modifications such as toxicokinetic assessments in offspring and additional endpoints. In our dataset, there were several cases where a well-designed enhanced PPND (n = 9) study justified not performing a JAS. This was primarily in relation to biologicals, where there are opportunities to maintain toxicologically relevant exposures in the offspring, allowing to evaluate safety relevant to paediatric patients.

### Timing of submission

When a JAS was already ongoing or completed during the PIP assessment, critical questions and requests for clarifications to justify the applicant’s approach were still made. However, the overall effect of the regulatory feedback on the need or design of a JAS in such cases was very limited, as alterations to the JAS plans were no longer an option. This has, in our opinion, led to the conduct of unnecessary studies and/or studies with suboptimal designs. Although it would be preferable to avoid such situations, the requests for clarification can be considered a learning opportunity for applicants. Submitting the PIP early in the development process would allow applicants to benefit from regulatory advice and ensure optimised and adequate study plans to support paediatric development.

### Waivers

Overall, in 38% of the applications the waiver age cut-off was lowered following PDCO assessment, which partly contributed to changes in the JAS plans (Figure 6). This observation suggests a lack of alignment between the applicants’ assessment and EMA considerations regarding the intend-to-treat paediatric population. It is crucial to clearly define the target indication and the intended paediatric population with supportive scientific arguments (12, 16). Recently, the guidance on the elements needed to assess the basis for a proposed waiver cut-off was updated and expanded in the new template of the PIP application Scientific Document to provide more context on how a relevant waiver cut-off can be established (17). In addition, previously agreed PIPs are publicly available, which the applicant can consult to ensure their waiver arguments are consistent with past PDCO decisions. Ensuring that the proposed waiver is largely in line with the final waiver further reduces the possibility of regulatory misalignment during the PIP assessment. However, if doubts remain regarding the adequate waiver age cut-off in the PIP, the applicants can request a pre-submission meeting to receive advice from EMA before formally submitting their application.

### Steps to encourage the use of WoE in planning paediatric development

Implementation of the ICH S11 guideline required raising awareness of the harmonised approach among medicine developers as well as health authorities. In the EU, this was addressed by updating the documents related to PIP assessment. The WoE slider and targeted questions (12) were included in the NcWP assessment form to remind assessors about the key aspects to consider when evaluating the need for a JAS within the non-clinical strategy. In addition, the EU assessors’ community has been offered training material on key aspects of the WoE assessment and principles of JAS study design in support of paediatric medicine development. Similarly, the Scientific Document template (B-F) of the PIP application was revised to include more elaborate guidance text, offering the applicant advice to use the ICH S11 methodology (17). The data collection for our study indicates that the quality of the WoE discussion from both assessors and applicants has indeed benefitted from the introduction of these measures.

### Lack of alignment between EMA and other health authorities

One of the objectives of ICH S11 was to promote international harmonisation of the non-clinical safety assessment of medicines intended for paediatric use. In a limited number of cases, a JAS was included in the opinion despite being considered unnecessary by the EMA, due to the apparent regulatory requirements of other health authorities. When a JAS was deemed necessary by another health authority, the applicant was sometimes prompted to undertake the study. Since the JAS was being conducted regardless, the PDCO included the study in the PIP opinion, and the additional data could support the MAA. Such cases are usually followed-up in a Paediatric Cluster meeting, where discussions on divergent approaches and data interpretations takes place between different global health authorities (18). Understanding the views of other health authorities is useful for the PIP application assessment. Therefore, submitting relevant meeting minutes or advice letters from other health authorities can help resolve any misalignment. Ideally, consulting health authorities in parallel before initiating definitive JAS studies would also help achieve a better level of global harmonisation.

### Limitations

This study had several limitations. While we attempted to generate a dataset that captured the early experience of applicants and EMA assessors after the implementation of ICH S11, some cases may have been missed or excluded (e.g., products in clock-stop) that could have been relevant to this analysis. Also, due to the high amounts of context specific data, some of this context may have been lost when we coded the data to allow aggregated analysis. Finally, while we believe this early period to be highly relevant to inform current practices, our analysis may not have captured all current trends in leveraging the WoE assessment.

### Future recommendations

There are a number of lessons learned from our retrospective review that can be used to enhance the implementation of the ICH S11 guideline. In some cases, applicants were requested by the EMA to remove non-core endpoints from the JAS study plan (Table 4). For instance, the retrospective analysis identified five cases where the removal of the FOB from the JAS was suggested (9) (Box 2, Case 1). According to ICH S11, the FOB or the modified Irwin test are considered to have low sensitivity in juvenile rodents, thus limiting their utility (12). This is partly due to the behavioral and physical variability between litters and test animals, which may be compounded by animal development (19). Instead, detailed timed CNS clinical observations in JAS should be considered as a key component of CNS assessment. These observations describe clinical signs throughout the study, including their time of onset and duration, allowing for the association of test-article related changes with respect to dosing. The need for specific behavioral tests, learning and memory assessments, or expanded neurohistopathology should be evaluated based on the WoE. The importance of a discussion on the role and expression of the target in the CNS, the extent of distribution across the BBB and potentially affected brain regions, to support the selection of the most adequate CNS endpoints, cannot be understated. Another example of a non-core and not recommended endpoint is the inclusion of mating cohorts. We observed multiple cases with such planned assessments, despite ICH S11 clearly stating that mating assessments are not generally recommended in a JAS. The same objectives can be accomplished by including other endpoints such as histopathology on male reproductive organs, or oestrous cyclicity and ovarian histology for rodent female reproductive concerns.

BOX 2 **Case studies—examples of rationales that led to modification of the JAS designs.**

**CASE 1 - Endpoints added and endpoints removed**

**Table:** 

**Proposed paediatric population:** 0yr - < 18yr	**Agreed population:** 0yr - < 18yr
**JAS proposed species:** rat	
**JAS proposed starting age**: PND 12	**Opinion JAS starting age**: PND 12
**Target organs of toxicity:** skin, bone marrow, lymphoid tissues, GI tract	
**Non-core endpoints proposed:** FOB, neurobehavioral testing, ophthalmologic examination	Final Endpoints*: bone histopathology, neurobehavioral testing

**Rationale:** Due to the medicine’s mechanism of action as a Janus Kinase (JAK) inhibitor, concerns arose regarding its potential effects on osteoblast differentiation and bone formation. Consequently, the NcWP recommended including bone histopathology as an additional endpoint alongside the core ICH S11 growth endpoint. In contrast, the absence of ophthalmologic effects in adult animals led to the exclusion of this endpoint for the JAS. Similarly, the FOB behavioural assessment was deemed unsuitable due to its low sensitivity in juvenile rodents and limited applicability. Nevertheless, the NcWP agreed with the proposed neurobehavioral testing (i.e. auditory startle response, locomotor activity, learning and memory tests) to address the CNS concerns identified based on the target biology and CNS effects observed in JAS from two other compounds in the same class.

*In addition to the core ICH S11 endpoints

**CASE 2 - Juvenile animal age at study start increased**

**Table:** 

**Proposed paediatric population:** 2yr - < 18yr	**Agreed population:** 2yr - < 18yr
**JAS proposed species:** rat	
**JAS proposed starting age**: PND 7	**Opinion JAS starting age**: PND 14
**Target organs of toxicity:** vasculopathy and inflammation in several tissues (bone, GI tract, cardiovascular and pulmonary system) Class effects on CNS.	

**Rationale:** Taking into consideration the age of the paediatric population from 2 to less than 18 years, the GI tract toxicity observed and the fact that the GI tract is functionally immature in rats for the first 2 weeks of life, it was recommended to start JAS at PND 14. In addition, the maturation status of the CNS in PND 14 old rats was considered more comparable to 2-year-old children than PND 7 old rats. Consequently, dosing initiation in slightly older rats (PND 7 -> PND 14) was expected to deliver more relevant data for characterising safety risks in the target population.

**CASE 3 - Juvenile animal age at study start decreased**

**Table:** 

**Proposed paediatric population:** 28 day- < 18yr	**Agreed population:** preterm -<18yr
**JAS proposed species:** rat	
**JAS proposed starting age**: PND 10	**Opinion JAS starting age**: PND 7
**Target organs of toxicity:** GI tract, liver, skeletal muscle necrosis and phospholipidosis in several organs.	

**Rationale:** As paediatric development was updated to include preterm neonates upon PDCO assessment, the WoE consideration changed. The CNS penetration of the drug was a concern due to BBB immaturity in the youngest target population. These concerns were further raised by phospholipidosis observed in Schwann cells with other similar products. Considering this, given the corresponding age in rats regarding neurogenesis, synaptogenesis, onset of myelination and brain plasticity it was recommended to start animal dosing at an earlier time point, at PND 7, to match the development stage of preterm neonates.

Another frequently discussed and subsequently modified aspect of JAS design is the age of the animals at study initiation (Table 4). The most frequently used JAS species is rat for which the ICH S11 guideline highlights that several organs are relatively immature in newborns pups compared to the human neonate. Despite this, neonatal rats at PND 7 are often proposed for JAS to support products developed for children from birth, which could be based on the interpretation of the simplified schematic in Figure A1 in ICH S11. However, as our knowledge of organ maturation across species has evolved, there are now arguments for delaying the start of dosing to PND 10/14 in rats, to better support human paediatric development from birth (9, 10, 20, 21). For example, recent knowledge of comparative brain development suggests that the CNS of a 7-day-old rat is less developed than that of a human newborn. This is evident in neurogenesis, synaptogenesis, myelination onset, and brain plasticity. Additionally, testing rat pups younger than 10 days old is not advisable based on the comparative development of other organs, such as the digestive system and kidneys, which are less mature at birth in rats than in humans. A benefit of dosing at a later stage is the avoidance of clinically irrelevant toxicities that may be observed in more immature animals (Case 2, Box 2). This aspect seems insufficiently addressed in ICH S11 and could benefit from further harmonisation. On the other hand, the decision on the animal age at dosing start is context specific. For instance, in one PIP, the first dose administration was advised to be changed to an earlier stage in development, from PND 10 to PND 7, to address potential toxicities in the revised target paediatric population, which included prematurely born infants in the final PIP (Case 3, Box 2).

### Shift in paradigm

Since the implementation of the ICH S11 guideline, EMA recommended the removal of JAS studies from roughly one third of those proposed by applicants as part of a PIP. This decision was largely driven by the lack of non-clinical findings which would be of specific concern for the intended paediatric population or a lack of concern for developing organs. EMA also recommended to change the JAS study design to improve translatability to the medicine’s clinical context of use and to better characterise safety risks in support of a paediatric development plan. The addition of a JAS, mostly motivated by a large degree of uncertainty about the potential toxicities of a medicine, was requested only in a small fraction of PIPs in which a JAS was not initially proposed (4/79, 5%).

Close to 70% of initially proposed JASs were either modified or removed from the final PIP opinion. This reflects a shift by regulators towards promoting the conduct of only fully optimised and informative JAS studies. This demonstrates a successful implementation of the WoE approach which scrutinised a variety of factors to ensure appropriate characterisation of medicine’s safety but also to avoid unnecessary or uninformative animal research. At the same time, it showcases an important need to receive regulatory feedback by the applicants.

In conclusion, the experience with the ICH S11 WoE approach led to a realisation that for the majority of development of pharmaceuticals for paediatric use, additional non-clinical testing in juvenile animals is not necessary. A thorough WoE discussion is needed, and regulatory interactions often facilitate a fully fleshed analysis leading to an informed decision on the non-clinical safety testing strategy.
