# Supplementary material for: From guideline to practice: three years of ICH S11 insights and recommendations
Source: Front Med (Lausanne). 2025 Feb 10;12:1537001. doi: 10.3389/fmed.2025.1537001 (PMC11847842; doi:10.3389/fmed.2025.1537001)
Supplement: Supplementary file 1 [file Data_Sheet_1.docx]

Supplementary Material

From Guideline to Practice: Three Years of ICH S11 Insights and Recommendations

# Supplementary Figures and Tables

For more information on Supplementary Material and for details on the different file types accepted, please see [here](https://www.frontiersin.org/guidelines/author-guidelines#supplementary-material).

## Supplementary Figures

##
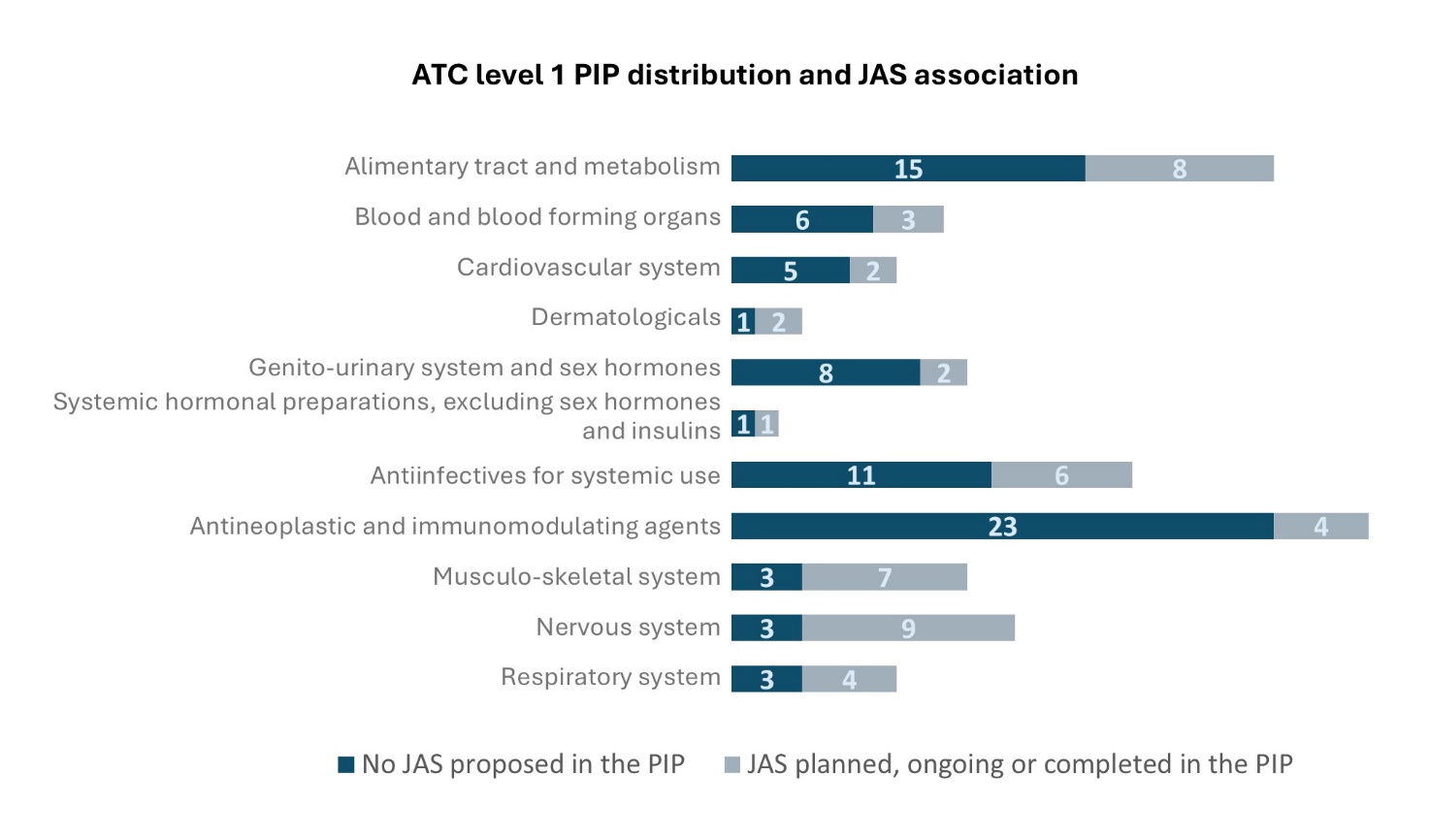


**Supplementary Figure 1.** Paediatric investigation plans (PIP) distribution per Anatomical Therapeutic Chemical (ATC) Classification level 1 and the completion status of juvenile animal studies (JAS).


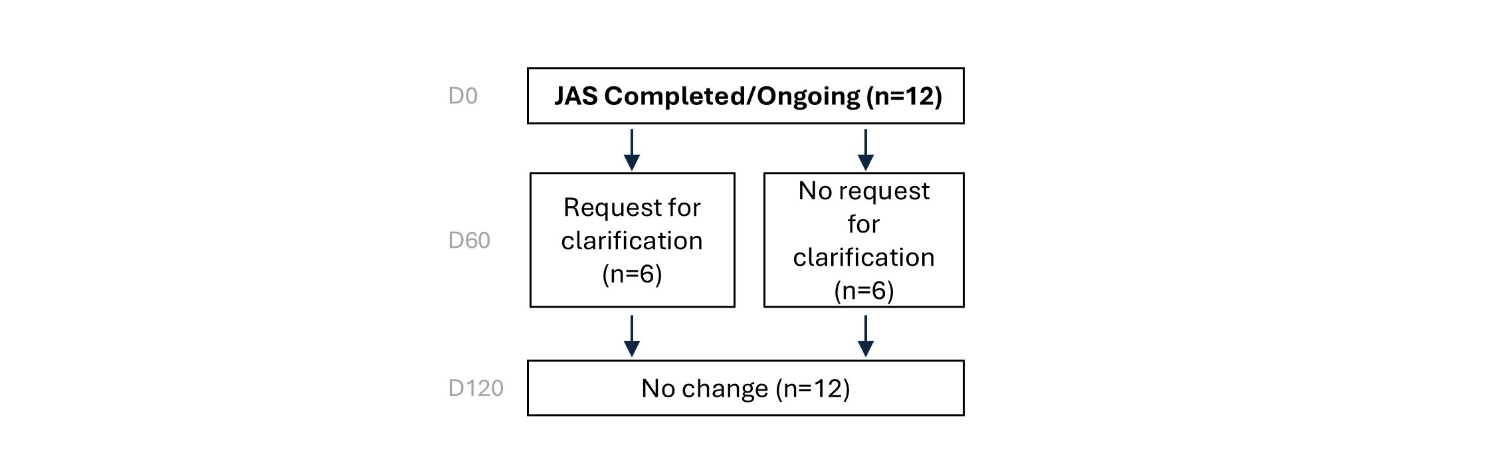
 **Supplementary Figure 2.** Overview of JAS removed, modified, or added based on EMA assessment on Day 120 in the subset of PIPs with JAS ongoing or completed at the time of PIP submission.

## Supplementary Tables

**Supplementary Table 1.** Factors supporting the absence of a JAS and requests for clarification (n=58)

| FINAL youngest intended patients | | | < 1 month  (n=14) | 1 month - < 6 month (n=6) | 6 month - < 24 month (n=13) | 2 year - < 12 year (n=23) | ≥ 12 year (n=2) | Total (n=58) |
| --- | --- | --- | --- | --- | --- | --- | --- | --- |
| Were requests for clarification partly due to waiver changed? (Day 60) | | Yes | 4 | 2 | 5 | 8 | 0 | 19 |
|  |  | No | 10 | 4 | 8 | 15 | 2 | 38 |
| Factors considered by the Applicant (Day 60) | Non-clinical data | |  |  |  |  |  |  |
|  | - Repeated-dose toxicity | | 13 | 4 | 13 | 21 | 2 | 52 |
|  | - Young/adolescent animals^1^ | | 4 | 1 | 8 | 14 | – | 26 |
|  | - Juvenile animals^2^ | | 1 | – | 1 | 1 | – | 3 |
|  | - FEED/EFD/(e)PPND | | 5 | 2 | 4 | 11 | – | 20 |
|  | - Off-target investigations | | 3 | 3 | 1 | 2 | 1 | 10 |
|  | - Safety pharmacology | | 1 | – | 1 | – | – | 2 |
|  | Clinical data | |  |  |  |  |  |  |
|  | - Adult | | 10 | 4 | 8 | 12 | 2 | 36 |
|  | - Paediatric | | – | 1 | – | – | – | 1 |
|  | Pharmacological properties | |  |  |  |  |  |  |
|  | - Discussion on PD-related effects or effects on developing organs | | 9 | 2 | 11 | 14 | 1 | 37 |
|  | - High selectivity for target | | 2 | 2 | 4 | 3 | 1 | 12 |
|  | Feasibility | |  |  |  |  |  |  |
|  | - JAS is not feasible | | 2 | 2 | 2 | 7 | – | 13 |
|  | Other | |  |  |  |  |  |  |
|  | - Step-wise approach in clinic | | 2 | 2 | 1 | 4 | – | 9 |
|  | - Literature data with similar drugs | | 3 | 1 | 2 | 2 | – | 8 |
|  | - Risk clinically manageable | | 3 | 1 | 2 | 1 | – | 7 |
|  | - Benefit/risk consideration | | 1 | 2 | 3 | 1 | – | 7 |
|  | - Clinical monitoring > JAS^3^ | | 1 | – | 3 | – | – | 4 |
|  | - Short-term treatment | | 1 | – | 2 | – | – | 3 |
|  | - Regulatory advice | | – | – | 1 | 1 | – | 2 |
| Requests for clarification (Day 60) | Discuss the relevance of presence or absence of non-clinical findings and/or theoretical PD-related effects on developing organs | |  |  |  |  |  |  |
|  | - Reproductive toxicity | | 1 | 2 | – | 2 | – | 5 |
|  | - CNS | | 1 | – | – | 3 | – | 4 |
|  | - Bone | | 1 | – | – | 1 | 1 | 3 |
|  | - GI tract | | – | 1 | – | – | – | 1 |
|  | - Pancreas, adipose tissue, lymphoid organs, submandibular glands | | – | – | – | 1 | – | 1 |
|  | - Micronuclei formation | | – | – | – | 1 | – | 1 |
|  | - Blood clotting, innate immunity induction | | – | – | – | 1 | – | 1 |
|  | - Relevance of species | | – | – | – | 1 | – | 1 |
|  | Provide/include additional data | |  |  |  |  |  |  |
|  | - (e)PPND results | | – | 1 | 1 | 2 | – | 4 |
|  | - Target expression | | 1 | – | 1 | – | – | 2 |
|  | - Exposure margins | | 1 | – | 1 | – | – | 2 |
|  | - Age of animals used in RDT | | – | – | – | 2 | – | 2 |
|  | - Method: off-target effect | | 1 | – | – | – | – | 1 |
|  | Iterative approach: re-consider a JAS when more data become available | | – | – | 1 | 1 | – | 2 |

^1^ = 4 – 9 week-old rats, 6 – 9 week-old mice, or 2 – 7 year-old monkeys; ^2^ = rats of PND 21 – 23; ^3^ = clinical monitoring was considered sufficient and a JAS considered not necessary.

**Supplementary Table 2**. Requests for clarification on Day 60: the absence of a JAS was challenged (n=22).

| ***FINAL*** youngest intended patients | | | | < 1 month  (n=8) | 1 month - < 6 month (n= 1) | 6 month - < 24 month (n=5) | 2 year - < 12 year (n=6) | ≥ 12 year (n=2) | **Total (n=22)** |
| --- | --- | --- | --- | --- | --- | --- | --- | --- | --- |
| Was the absence of a JAS challenged partly due to a changed waiver? (Day 60) | | | Yes | 2 | 1 | 3 | 2 | 2 | 10 |
|  |  |  | No | 6 | 0 | 2 | 4 | 0 | 12 |
| Requests on non-clinical data (Day 60) | Clinical relevance (absence) of effects on developing organs: | | |  |  |  |  |  |  |
|  | - CNS | | | 3 | – | 1 | 5 | – | 9 |
|  | - Reproductive organs | | | – | – | 1 | 2 | 1 | 4 |
|  | - Bone | | | – | – | – | 1 | 1 | 2 |
|  | - Liver | | | – | 1 | 1 | – | – | 2 |
|  | - Kidney | | | – | – | 1 | – | – | 1 |
|  | - Blood vessel | | | 1 | – | – | – | – | 1 |
|  | - Muscle | | | – | – | – | 1 | – | 1 |
|  | - Lymphoid organs | | | – | – | – | 1 | – | 1 |
|  | - Adrenals | | | – | – | – | 1 | – | 1 |
|  | - Heart | | | – | – | – | 1 | – | 1 |
|  | MoA of non-clinical findings: | | |  |  |  |  |  |  |
|  | - CNS | | | 2 | – | 1 | 2 | – | 5 |
|  | - Inflammatory adventitial findings | | | 1 | – | – | – | – | 1 |
|  | - Heart | | | – | – | – | 1 | – | 1 |
|  | Results/relevance of PPND study | | | 1 | 1 | 1 | – | – | 3 |
|  | Off-target investigation | | | 2 | – | – | 1 | – | 3 |
| Requests on pharmacological properties (Day 60) | MoA of compound | | |  |  |  |  |  |  |
|  | - CNS | | | 1 | – | – | – | – | 1 |
|  | - Regulatory T cells | | | – | – | 1 | – | – | 1 |
|  | - Organic acid metabolism | | | 1 | – | – | – | – | 1 |
|  | - Knowledge of target | | | – | – | 1 | – | – | 1 |
|  | Role of the target/PD in development | | |  |  |  |  |  |  |
|  | - CNS | | | 1 | – | 1 | – | – | 2 |
|  | - Liver | | | – | 1 | – | – | – | 1 |
|  | - Urinary system | | | 1 | – | – | – | – | 1 |
|  | - Hematopoietic system | | | 1 | – | – | – | – | 1 |
|  | - Skeletal system | | | 1 | – | – | – | – | 1 |
| Other requests (Day 60) | Clinical monitoring plan | | |  |  |  |  |  |  |
|  | - CNS effects | | | 1 | – | – | – | – | 1 |
|  | - Anti-lipid and anti-PEG antibodies | | | 1 | – | – | – | – | 1 |
|  | - As an alternative to JAS | | | – | 1 | – | – | – | 1 |
|  | Literature data with similar drugs | | | 1 | – | – | 1 | 2 | 3 |
|  | Relevance of data with surrogate molecule | | | 1 | – | – | – | – | 1 |
|  | Predictivity of species | | | 1 | – | 1 | – | – | 2 |
|  | Receptor specificity vs other similar drugs | | | – | – | – | – | 1 | 1 |
|  | Feasibility of JAS | | | 1 | – | – | – | – | 1 |
| NcWP/PDCO conclusion on Day 120 | | No change | | 7 | 1 | 4 | 4 | 2 | 18 |
|  |  | JAS added | | 1 | 0 | 1 | 2 | 0 | 4 |

**Supplementary Table 3**. Factors supporting the need for a JAS considered by the Applicant (n=17).

| FINAL youngest intended patients | | | < 1 month  (n=3) | 1 month - < 6 month (n=2) | 6 month - < 24 month (n=2) | 2 year - < 12 year (n=10) | Total (n=17) |
| --- | --- | --- | --- | --- | --- | --- | --- |
| Were there waiver changes? (Day 60) | | Yes | 1 | 1 | 1 | 4 | 7 |
|  |  | No | 2 | 1 | 1 | 6 | 10 |
| Factors related to available data with the molecules and PD | Non-clinical data | |  |  |  |  |  |
|  | - Repeated-dose toxicity | | 3 | 1 | 1 | 8 | 13 |
|  | - - Young/adolescent animals | | – | – | – | 4 | 4 |
|  | - FEED/EFD/(e)PPND | | – | – | – | 4 | 4 |
|  | - Off-target investigation | | 1 | – | – | 1 | 2 |
|  | - Safety pharmacology | | 1 | – | – | 3 | 4 |
|  | Clinical data | |  |  |  |  |  |
|  | - Adult | | 2 | – | – | – | 2 |
|  | - Adolescent | | 1 | – | – | – | 1 |
|  | Pharmacological properties | |  |  |  |  |  |
|  | - Discussion on PD-related effects or effects on developing organs | | 2 | 1 | 1 | 10 | 13 |
| Other factors | No factor identified/unclear rationale | | 1 | 1 | 1 | 1 | 4 |
|  | Regulatory advice | | 1 | – | – | 2 | 3 |
|  | Literature data with similar drugs | | – | – | – | 2 | 2 |
|  | Effects cannot be monitored in clinic | | – | – | – | 1 | 1 |
| The added value of a JAS considered by the Applicant | JAS was considered required to characterize effects on: | |  |  |  |  |  |
|  | CNS | | 1 | 1 | – | 3 | 5 |
|  | Reproductive organs | | – | – | 1 | 2 | 3 |
|  | Endocrine system | | – | – | – | 3 | 3 |
|  | Bone marrow | | 1 | – | 1 | 1 | 3 |
|  | GI tract | | 1 | 1 | 1 | – | 3 |
|  | Bone | | – | – | 1 | 1 | 2 |
|  | Hepatobiliary system | | – | 1 | 1 | – | 2 |
|  | Multiorgan phospholipidosis | | 1 | – | – | 1 | 2 |
|  | Lymphoid organs | | – | – | 1 | 1 | 2 |
|  | Kidney | | – | – | 1 | – | 1 |
|  | Skin | | – | – | 1 | – | 1 |
|  | Skeletal muscle | | 1 | – | – | – | 1 |
|  | Pulmonary system | | – | – | – | 1 | 1 |
|  | Cardiovascular system | | – | – | – | 1 | 1 |
|  | JAS was considered required to define exposure margin | | – | – | – | 1 | 1 |

**Supplementary Table 4.** Modifications to the JAS design in the final opinion (n=12)

| FINAL youngest intended patients | | | < 1 month  (n=2) | 1 month - < 6 month (n=2) | 6 month - < 24 month (n=1) | 2 year - < 12 year (n=7) | Total (n=12) |
| --- | --- | --- | --- | --- | --- | --- | --- |
| Was the waiver lowered? (Day 60) | | Yes | 1 | 1 | 1 | 2 | 5 |
|  |  | No | 1 | 1 | 0 | 5 | 7 |
| Modifications to the youngest starting age of animals (rat only) | Age of animals increased | |  |  |  |  |  |
|  | PND4 → PND14 | | – | – | 1 | – | 1 |
|  | PND7 →PND10 | | – | 1 | – | – | 1 |
|  | PND7→PND14 | | – | 1 | – | 1 | 2 |
|  | Age of animals decreased | |  |  |  |  |  |
|  | PND10 → PND7 | | 1 | – | – | – | 1 |
|  | PND28 → PND21 | | – | – | – | 1 | 1 |
| Modifications to the endpoints | Endpoints removed | |  |  |  |  |  |
|  | CNS assessment | | 1 |  | – | 1 | 2 |
|  | Ophthalmologic exams | | 1 | – | – | 1 | 2 |
|  | Bone density assessment | | – | 1 | – | – | 1 |
|  | Reproductive assessment | | 1 | – | – | – | 1 |
|  | Endpoints added | |  |  |  |  |  |
|  | CNS assessment^1^ | | 1 | 2 | – | 3 | 6 |
|  | Bone histology, density, biomarker for formation and resorption | | 1 | – | – | 1 | 2 |
| Modifications to the dosing or recovery period | Dosing duration increased | |  |  |  |  |  |
|  | PND63→PND90 | | – | – | – | 1 | 1 |
|  | PND35→PND70 | | – | – | – | 1 | 1 |
|  | Recovery period extended | | – | – | – | 1 | 1 |
| Modifications to the timepoints of measurement and dose group | Timepoints for CNS endpoints measurement added | |  |  |  |  |  |
|  | At CNS peak exposure | | – | – | – | 1 | 1 |
|  | In or at the end of recovery | | – | – | – | 2 | 2 |
|  | During dosing | | – | – | – | 1 | 1 |

PND = postnatal day; FOB = functional observational battery; DEXA = Dual Energy X-ray Absorptionmetry; 1 = expanded neurohistopathology, detailed clinical evaluation, drug concentration in brain tissue, cerebrospinal fluid composition.
